# Regulation of the Intestinal Barrier Function by Host Defense Peptides

**DOI:** 10.3389/fvets.2015.00057

**Published:** 2015-11-23

**Authors:** Kelsy Robinson, Zhuo Deng, Yongqing Hou, Guolong Zhang

**Affiliations:** ^1^Department of Animal Science, Oklahoma State University, Stillwater, OK, USA; ^2^Hubei Key Laboratory of Animal Nutrition and Feed Science, Wuhan Polytechnic University, Wuhan, China

**Keywords:** host defense peptides, barrier function, tight junction, gut health, innate immunity

## Abstract

Intestinal barrier function is achieved primarily through regulating the synthesis of mucins and tight junction (TJ) proteins, which are critical for maintaining optimal gut health and animal performance. An aberrant expression of TJ proteins results in increased paracellular permeability, leading to intestinal and systemic disorders. As an essential component of innate immunity, host defense peptides (HDPs) play a critical role in mucosal defense. Besides broad-spectrum antimicrobial activities, HDPs promotes inflammation resolution, endotoxin neutralization, wound healing, and the development of adaptive immune response. Accumulating evidence has also indicated an emerging role of HDPs in barrier function and intestinal homeostasis. HDP deficiency in the intestinal tract is associated with barrier dysfunction and dysbiosis. Several HDPs were recently shown to enhance mucosal barrier function by directly inducing the expression of multiple mucins and TJ proteins. Consistently, dietary supplementation of HDPs often leads to an improvement in intestinal morphology, production performance, and feed efficiency in livestock animals. This review summarizes current advances on the regulation of epithelial integrity and homeostasis by HDPs. Major signaling pathways mediating HDP-induced mucin and TJ protein synthesis are also discussed. As an alternative strategy to antibiotics, supplementation of exogenous HDPs or modulation of endogenous HDP synthesis may have potential to improve intestinal barrier function and animal health and productivity.

## Introduction

The gastrointestinal (GI) tract is lined by a single layer of epithelial cells that serve to facilitate digestion and absorption of nutrients and also act as a barrier to invading microorganisms, toxins, and dietary antigens. Intestinal barrier function is achieved through coating of the epithelial cells with a mucus layer and the formation of a selectively permeable barrier across and between epithelial cells (1). The mucus layer consists primarily of mucin glycoproteins that are secreted by goblet cells, functioning as a physical barrier between the luminal contents and the host and also to facilitate nutrient digestion and absorption (2). However, the primary barrier function of the GI tract resides with epithelial cells, which transport water, ions, and macromolecules through either of two routes, i.e., the transcellular and paracellular pathways (1, 3, 4). The transcellular pathway refers to the movement of small molecules through epithelial cells either by active or passive transport, whereas the paracellular pathway refers to the diffusion of water, macromolecules, and immune cells between epithelial cells. In the presence of intact epithelial cells, the paracellular pathway dictates the intestinal permeability and is regulated by inter-epithelial connections known as tight junctions (TJs) (1, 3, 4).

Maintenance of mucin and TJ assembly ensures proper absorption and transport of nutrients, water, and electrolytes, while shielding the host from pathogens, toxins, intestinal microbiota, and dietary antigens. Disruption of the mucus layer and TJ complex, on the other hand, results in an increase in intestinal permeability, followed by heightened bacterial translocation, inflammation, and possibly the onset of various enteric and systemic disorders (1, 3, 4). In livestock production, impaired intestinal barrier function leads to reduced animal health and growth performance (5, 6). Therefore, it is critically important to understand how the intestinal barrier function is maintained and regulated in order to achieve optimal animal health and productivity.

Host defense peptides (HDPs), also known as antimicrobial peptides, are an important component of the animal innate immune system, and a majority of HDPs are expressed on mucosal surfaces, including the GI tract (7, 8). With potent antimicrobial and immunomodulatory activities, HDPs exert a pleiotropic effect on innate adaptive immune responses (9–11). Recent research has further shed light on the direct involvement of epithelial HDPs in regulating intestinal mucin and TJ protein expression and microbiota composition. The focus of this review is to summarize the latest advances regarding the emerging role of HDPs in maintaining intestinal barrier and homeostasis with a goal of exploring HDP-based therapies to improve gut health and performance of food-producing animals.

## Host Defense Peptides: A Critical Component of Innate Immunity

A variety of HDPs with direct antimicrobial activities are produced by host cells in response to infections. Among them are several major families found in vertebrate species such as defensins, cathelicidins, the S100 family, the RNase A superfamily, regenerating islet-derived III (REGIII) C-type lectins, and peptidoglycan-recognition proteins (12, 13). Defensins are primarily identified by three conserved disulfide bridges that form several antiparallel β-sheets due to the presence of multiple cysteine residues (14). Based on the spacing pattern of six cysteines, vertebrate defensins are further categorized into three subfamilies, including α-, β-, and θ-defensins. While β-defensins are present in all vertebrate animals, α-defensins are found in most but not all mammals, and θ-defensins only exist in primates (14). Cathelicidins are structurally recognized by the highly conserved cathelin domain found in the precursor that is cleaved off to release the biologically active peptides adopting a variety of structures such as α-helix (15).

The S100 family proteins are 9–14 kDa in mass containing two highly conserved Ca^2+^-binding EF-hand domains that are separated by four α-helical domains with a variable C-terminal region (16, 17). The RNase A superfamily are characterized by the presence of 6–8 conserved cysteines forming distinct disulfide bridges, together with two invariantly spaced histidines and a lysine (18, 19). The REGIII family proteins are a group of soluble C-type lectins with a conserved carbohydrate-recognition domain that binds to sugars in a Ca^2+^-dependent manner (20). Peptidoglycan-recognition proteins constitute a family of phylogenetically conserved host defense molecules with a PGRP domain that binds to bacterial peptidoglycans through specific interactions with the muramyl-tripeptide fragments (21).

### Expression of HDPs

Six α-defensins (22) and a minimum of 39 β-defensins (23) have been reported in humans. The genomes of cattle and pigs encode at least 57 and 29 β-defensin genes, respectively (24, 25), while the chicken genome harbors a total of 14 β-defensin genes (26, 27), with no α-defensins being found in cattle, pigs, or chickens. All β-defensin genes are located in tandem in a single genomic region in the chicken (26) and are expanded to 4–5 different clusters in humans, cattle, and pigs (23–25). Interestingly, all human α-defensin genes form a single cluster within a β-defensin gene cluster (22), suggesting that α-defensins likely diverged from β-defensins. While four human α-defensins (HNP1–4) are abundantly present in neutrophil granules, the other two α-defensins (HD5 and HD6) are specific to Paneth cells in the crypts of the human small intestinal tract (14). On the other hand, a majority of β-defensins are expressed in a wide range of cell types, particularly the epithelial cells lining the skin, GI, respiratory, and urogenital tracts of all livestock species as well as humans (14).

A single cathelicidin known as LL-37, CAMP, or hCAP-18 is present in humans (28) and four cathelicidins are reported in chickens (29, 30). In cattle and pigs, 10 and 11 cathelicidins have been identified, respectively (31, 32). All cathelicidin genes are located in a syntenic chromosomal region in vertebrate species. Expressions of cathelicidins are widespread with abundant presence in neutrophil granules as well as various epithelial mucosal surfaces of cattle, pigs, and humans. Four chicken cathelicidins are similarly expressed in a broad range of tissues as well as in heterophils (29, 30, 33, 34), which are equivalent to neutrophils in mammals. Additionally, chicken cathelicidin-B1 is highly expressed in M cells of the bursa of Fabricius (30), a type of specialized epithelial cells involved primarily in antigen transportation from the intestinal lumen to submucosal immune cells (35).

The S100 family members have been found in all vertebrates (17). A total of 21 S100 proteins are present in humans, with 17 members clustered in the same 2-Mb region on chromosome 1q21 (17). The tissue expression pattern of S100 proteins is unique and isoform specific (16). For example, S100A7 (also known as psoriasin) isolated initially from the skin of psoriatic patients is mainly expressed in the skin and breast tissues, whereas the heterodimer S100A8/S100A9, or calgranulin A/B, is expressed in keratinocytes, neutrophils, monocytes, and macrophages.

### Biological Functions of HDPs

Host defense peptides are an integral part of the innate immune system. Historically, HDPs are known for their ability to function as natural antibiotics with broad-spectrum antimicrobial activities against Gram-negative and Gram-positive bacteria, fungi, viruses, protozoa, and even cancerous cells (7, 36). Human LL-37 and α- and β-defensins are all capable of killing a broad spectrum of pathogens (7, 36). All four chicken cathelicidins have been demonstrated to be active at low micromolar concentrations against both Gram-positive and Gram-negative bacteria, including antibiotic-resistant strains (29, 30, 37–39). Several chicken β-defensins are also potent against a range of human and zoonotic pathogens (40–42). Similarly, β-defensins and cathelicidins in the cattle and pigs are broadly active against multiple pathogens as well (32).

Because of the cationic and amphipathic properties associated with a majority of HDPs, they kill bacteria primarily through disruption of cell membranes and/or interaction with intracellular macromolecules (43). A net positive charge allows HDPs to bind to negatively charged phospholipid groups on the bacterial membrane through electrostatic interactions. The amphipathic nature of HDPs facilitates their insertion into target cellular membranes allowing them to disrupt its integrity. Multiple models of membrane disruption, such as “barrel-stave”, “carpet,” or “toroidal-pore” models, have been proposed (43). Intracellularly, certain HDPs are also capable of inhibiting protein, DNA and RNA synthesis, or binding to specific targets (43). Because of their primary membrane-lytic activities, HDPs are generally equally active among drug-resistant and -susceptible pathogens. It is conceivably more difficult for pathogens to develop resistance to HDPs, although certain bacteria have developed mechanisms to resist their action in order to infect and colonize the hosts (44). It appears that commensal bacteria are generally resistant to the action of the constitutively expressed HDPs, but sensitive to certain inducibly expressed HDPs in the human intestinal tract (45). However, the mechanism by which commensal and probiotic bacteria show a reduced sensitivity to HDPs remains elusive.

**Figure 1:**
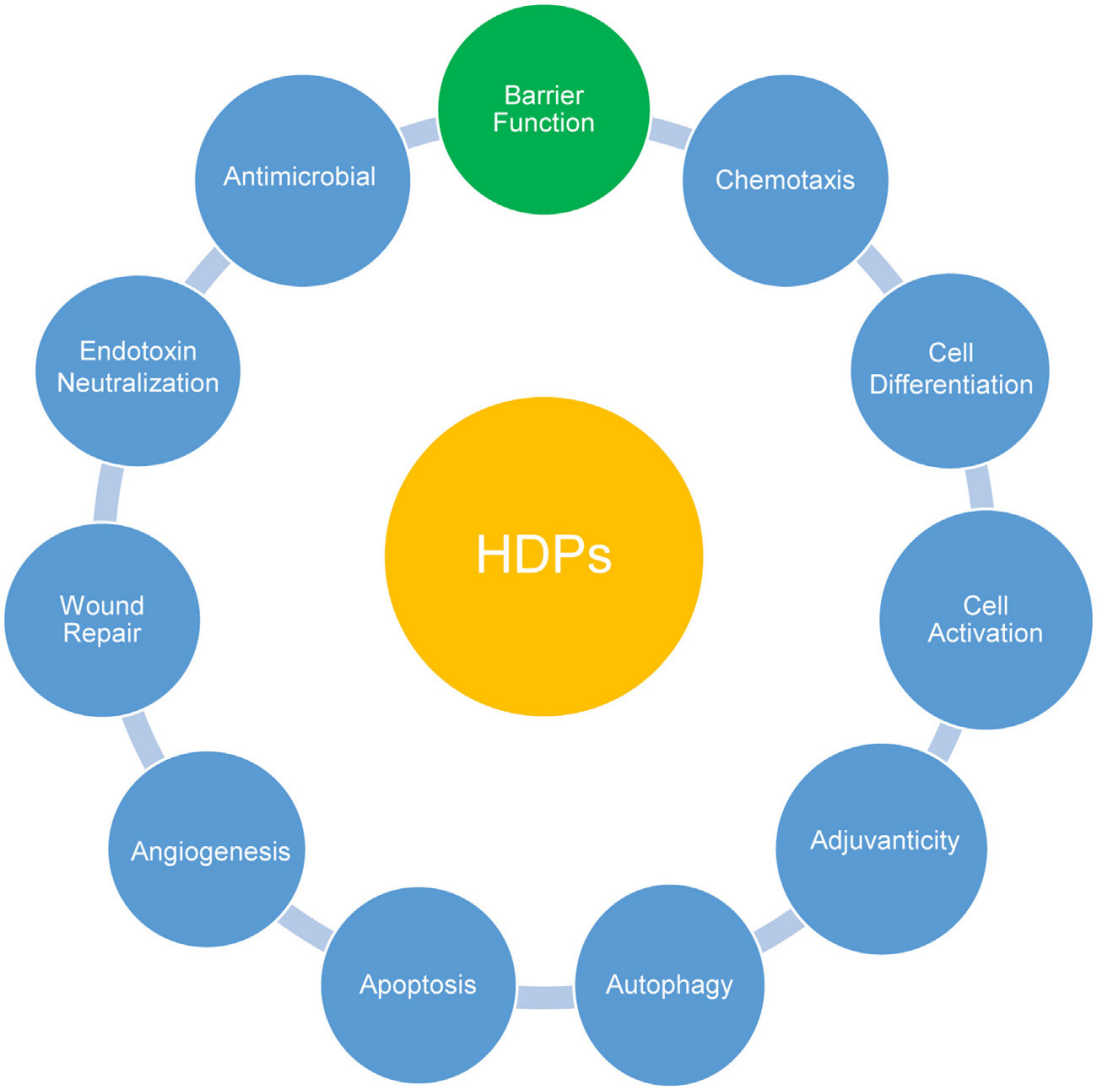
**Multifunctional roles of host defense peptides (HDPs)**. Besides direct antimicrobial activities, HDPs actively participate in systemic and mucosal epithelial defense by modulating a range of host innate and adaptive immune responses as indicated. Recent accumulating evidence has highlighted a direct involvement of HDPs in improving intestinal and epidermal barrier function.

Besides their antimicrobial activity, HDPs are involved in the modulation of innate and adaptive immune responses (10, 11) (Figure 1). Many human HDPs have been shown to promote the recruitment of neutrophils or monocytes and suppress proinflammatory response. Human HDPs also induce the differentiation and activation of macrophages and dendritic cells. Additionally, human cathelicidin LL-37 facilitates the resolution of inflammation by promoting re-epithelialization and wound healing as well as autophagy and apoptosis (10, 11). Three chicken cathelicidins known as fowlicidin 1–3 bind to bacterial lipopolysaccharides (LPS) directly with a strong capacity to neutralize LPS-induced production of inflammatory cytokines in macrophage cells (37–39). Furthermore, chicken fowlicidin-1 is chemotactic to neutrophils, but not monocytes or lymphocytes (46). Fowlicidin-1 also activates macrophages by inducing modest synthesis of inflammatory cytokines and chemokines and further potentiates the antibody response if co-administered with a model antigen (ovalbumin) in mice (46). Importantly, a single application of fowlicidin-1 is not only able to protect animals from an established infection (47) but also to prevent the disease beyond a 2- to 4-day window (46) in a murine model of methicillin-resistant *Staphylococcus aureus* (MRSA) infection (46).

Among 14 bovine β-defensins examined, three (BNBD3, BNBD9, and EBD) are chemotactic to immature monocyte-derived dendritic cells (48). Porcine cathelicidin PR-39 is also capable of inhibiting phagocyte NADPH oxidase activity and attenuating myocardial ischemia–reperfusion injury (49) by blocking the assembly of the enzyme complex through binding to p47phox, a cytosolic component of the NADPH oxidase (50). PR-39 accelerates wound repair by inducing syndecans (51). Furthermore, PR-39 facilitates angiogenesis and formation of functional blood vessels by inhibiting the ubiquitin–proteasome-dependent degradation of hypoxia-inducible factor (HIF)-1α (52). Several porcine cathelicidins also help with the update of bacterial DNA and subsequent activation of dendritic cells (53). HDPs with potent antimicrobial activity and the ability to modulate innate and adaptive immunity are, therefore, being actively exploited as novel antibiotics.

Additionally, recent emerging evidence has highlighted the beneficial effect of HDPs on mucosal barrier permeability by directly regulating mucin and TJ protein expression and shaping microbiota composition. This emerging role of HDPs in intestinal barrier function and homeostasis will be the focus of this review.

## Mucus Layer: A Layer of Intimate Protection for Mucosal Surface

**Figure 2:**
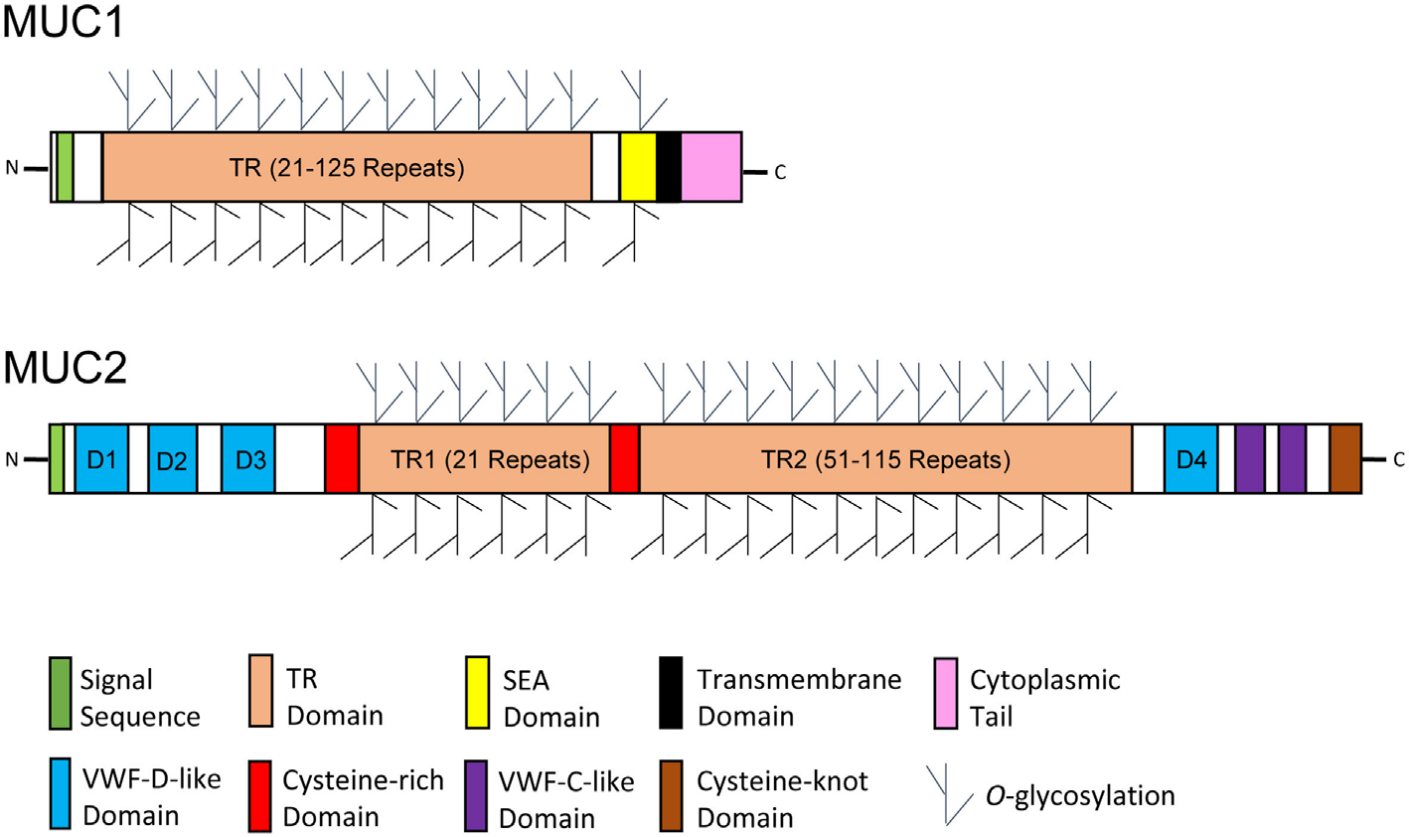
**Schematic diagrams of glycosylated mucin monomers**. Representative membrane-bound and secreted mucins are exemplified by mucin 1 (MUC1) and MUC2, respectively. The protein backbone of mucins is characterized by the presence of an excessive number of tandem repeat (TR) sequences. Mucins are heavily *O*-glycosylated through enriched threonine and serine residues in the TR domain. A transmembrane domain, a cytoplasmic tail, and a sea urchin sperm protein, enterokinase, and agrin (SEA) domain are unique to most membrane-bound mucins, whereas the presence of several von Willebrand factor (VWF) domains and a C-terminal cysteine-knot domain is specific to a majority of secreted mucins. The diagrams were modified primarily from reference (56).

An intact mucus layer that is composed primarily of secreted mucins plays a critical role in maintaining the intestinal barrier function (54, 55). Mucins are large, highly glycosylated proteins ranging from 0.5 to 20 MDa. Synthesized and released by goblet cells, mucins function to coat the mucosal surface to facilitate the passage of substances, maintain proper cell hydration, act as a permeable barrier for the exchange of gas and nutrients, and also protect the epithelial cells from invading pathogens and toxins (54, 55). Structurally, a hallmark of all mucin protein backbones is the presence of 1–5 tandem repeat (TR) domains, which consist of an excessive number of identical or nearly identical TR sequences rich in serine, threonine, and proline residues (56) (Figure 2). The TR domain is heavily glycosylated because of attachment of oligosaccharides to serine and threonine through *O-*linked glycosylation, giving rise to 50–80% glycans in mass. Saturated sugar coating is beneficial to increase the water-holding capacity and the resistance of mucins to proteolytic cleavage.

In humans, the mucin family consists of up to 20 members, including both secreted and membrane-bound forms. Secreted mucins form homo-oligomeric, gel-like structures constituting the mucosal layer, while the membrane-bound mucins are part of the epithelial glycocalyx that are involved in cell signaling and interactions with the environment without forming gel or oligomerization (57). Secreted human mucins include MUC2, -5AC, -5B, -6, -7, and -19, and the transmembrane mucins consist of MUC1, -3, -4, -12, -13, -15, -16, -17, and -20 (54, 55). Structurally, most secreted mucins are unique in the presence of multiple von Willebrand factor (VWF) domains and a C-terminal cysteine-knot domain, while a majority of membrane-bound mucins specifically consist of a transmembrane domain, a cytoplasmic tail, 1–2 epidermal growth factor (EGF)-like domains, and a sea urchin sperm protein, enterokinase, and agrin (SEA) domain (56, 58) (Figure 2). The VWF domains and cysteine-knot domain of secreted mucins are responsible for formation of higher-order structures through oligomerization, while the EGF-like and SEA domains of membrane-bound mucins mediate signaling transduction and cleavage of the extracellular portion of mucins, respectively (58). Among all secreted mucins, MUC2 is the most abundant in the human small intestine and colon, and MUC5AC is predominant in the stomach. Structurally, similar mucins have been found in most other vertebrate species, including cattle, pigs, and chickens (59–62).

The mucus layer formed by secreted mucins varies in composition along the GI tract. The stomach and large intestine consist of two distinct mucus layers: a “loose” outer layer and a “thick” inner layer (2). The inner layer closest to epithelial cells is densely packed and holds firmly to the cells. The inner mucus layer is largely free of bacteria, providing a sterile protective environment for the epithelium. The outer mucus layer is much more soluble due to proteolytic cleavages that allow the mucus layer to expand without disrupting mucin polymers. This outer layer provides a habitat for commensal bacteria to bind via specific adhesins and to thrive via breaking down the mucin glycans as a food source. Specificity of bacteria for different glycans is speculated to be important for developing species-specific microbiota (2).

Altered expression or glycosylation of mucins is often associated with intestinal barrier dysfunction (57). For example, Muc2 deficiency in mice causes increased permeability, gross bleeding, spontaneous development of inflammation in the GI tract, as well as severe growth retardation (63, 64). Muc1- or Muc2-deficient mice become more prone to infections with *Campylobacter jejuni*, *Helicobacter pylori*, *Salmonella enterica* serovar Typhimurium, and *Citrobacter rodentium* (64–67). Moreover, mice lacking the enzyme, β1,3-*N*-acetylglucosaminyltransferase that synthesizes *O*-glycans on mucins, exhibit a thinner mucus layer showing an enhanced susceptibility to enteric bacterial infections (67) and dextran sodium sulfate (DSS)-induced colitis (68). Additionally, significantly reduced expressions of multiple mucins such as MUC1, MUC3, MUC4, and MUC5B are observed in ileal mucosa of Crohn’s disease (CD) patients (69), although the expression changes of mucins are less clear in ulcerative colitis (UC) patients (70).

## Tight Junctions: Gate Guards for Border Protection

**Figure 3:**
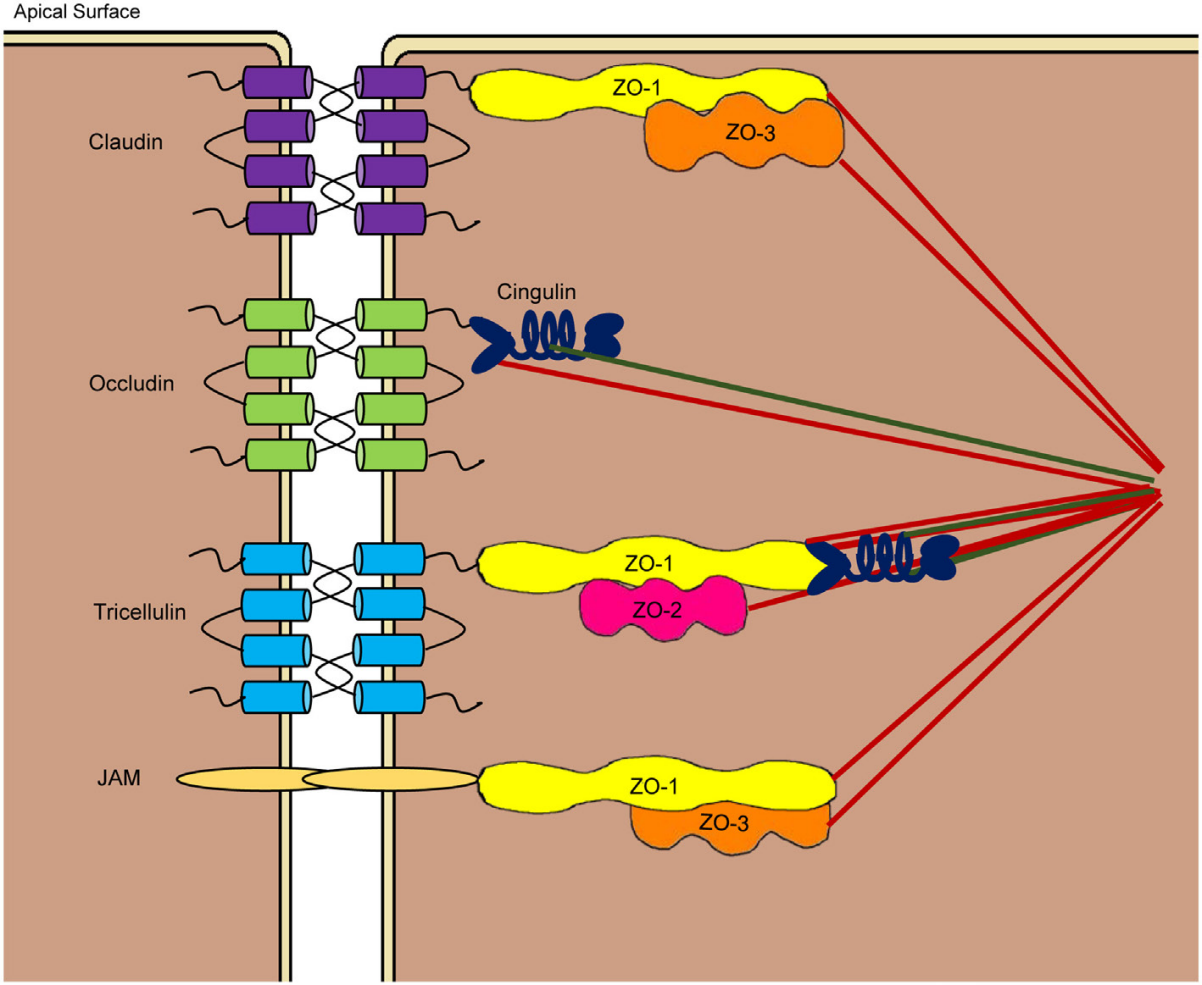
**Schematic drawing of tight junction structures at the apicolateral membranes of the paracellular space**. Tight junctions are comprised of transmembrane proteins such as claudins, occludin, junctional adhesion molecules (JAM), and tricellulin as well as cytoplasmic plaque proteins such as three zonula occludens (ZO) proteins and cingulin. Claudins, occludin, and tricellulin consist of four transmembrane domains, while JAMs have a single transmembrane domain. Through largely homophilic interactions, neighboring cells are sealed at the apicolateral region. Cytoplasmic proteins link the tight junction assembly to the cytoskeleton to control the opening of paracellular pores.

The intestinal epithelium is made up of several different cell types organized into crypts and villi. These include intestinal epithelial stem cells, enterocytes, and secretory cells, such as Paneth, goblet, and enteroendocrine cells (71). Intestinal stem cells give rise to all other epithelial cell types, while enterocytes primarily function in nutrient absorption with the ability to synthesize and release HDPs and mucins. Paneth and goblet cells are major producers of HDPs and mucins, respectively, while enteroendocrine cells have a primary role of secreting numerous hormones that act as regulators of digestive function (71). All intestinal epithelial cells are linked at lateral membranes through formation of three major types of junctional complexes, i.e., TJs, adherens junctions, and desmosomes (1, 3, 72). Collectively, they form a virtually impermeable seal to the paracellular space. Besides the barrier function, these junctional complexes maintain cell polarity by separating the apical from basolateral membranes. TJs are multi-protein complexes located at the most apical end of the lateral membrane. The TJ assembly is composed of both transmembrane and cytoplasmic plaque proteins that interact directly with the cytoskeleton (Figure 3). Among all three major junctional complexes, only TJs have the ability to control the selective paracellular permeability for ions, water, and other small molecules (1, 3, 72). Therefore, TJs are the major determinant of mucosal epithelial permeability.

### Tight Junction Structures

Among the proteins involved in TJ assembly, claudins, occludin, junctional adhesion molecules (JAM), and tricellulin are the major transmembrane proteins that constitute a selective paracellular barrier, whereas zonula occludens (ZO) and cingulin are the main cytoplasmic plaque proteins located at the peripheral membrane (1, 3, 72). All TJ proteins are highly conserved in vertebrate species. Claudins are a large family of small proteins of 21–34 kDa that make up the backbone of the TJ structure, with at least 26 members reported in humans (73). Remarkably, each claudin shows a unique tissue expression pattern with varied levels of expression in different segments of the GI tract. Occludin (65 kDa) was the first transmembrane TJ protein identified (74), with no homologs being found (75). On the other hand, the JAM family is comprised of three classical members (JAM-1, -2, and -3) and four related molecules (JAM-4, JAM-L, CAR, and ESAM) at ~40 kDa each (76). Tricellulin is a 64-kDa protein located preferentially at tricellular junctions, although it is also present in bicellular junctions (77). Tricellulin shares 32% identity in the amino acid sequence with the C-terminal tail of occludin. ZO proteins belong to the family of membrane-associated guanylate kinase (GUK) homologs that include three members, i.e., ZO-1 (~220 kDa), ZO-2 (~160 kDa), and ZO-3 (~130 kDa) (78), whereas cingulin is an ~140-kDa protein that links ZO proteins to the actin cytoskeleton (79).

**Figure 4:**
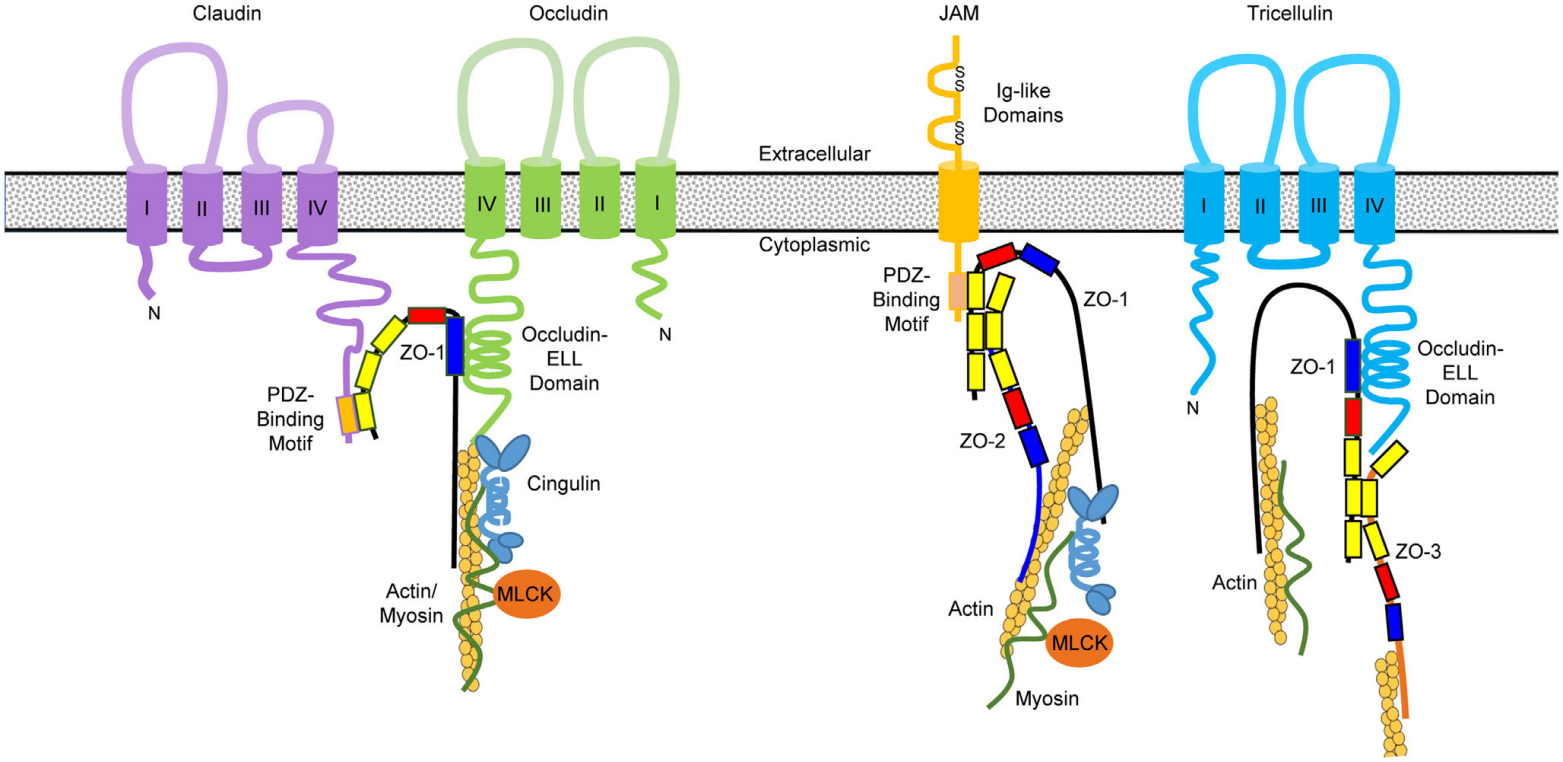
**Domain structures of primary tight junction proteins and their interactions**. Claudins consist of four transmembrane domains with a PDZ-binding motif at the C-terminal tail, which interacts with the first PDZ domain (in yellow) of zonula occludens (ZO)-1. The GUK domain (in blue) of ZO-1 binds to the occludin–ELL domain at the C-terminal tail of occludin and possibly tricellulin as well. Junctional adhesion molecules (JAM) contain a single transmembrane domain, two immunoglobulin (Ig)-like domains in the extracellular region, and a PDZ-binding motif at the C-terminal tail that interacts with the third PDZ domain of ZO-1. ZO molecules form heterodimers through interactions at their second PDZ domain. The C-terminal segment of ZO proteins binds directly to actin filaments as well as the globular head of cingulin that interacts with myosin through its rod domain. The globular head of cingulin also binds to the C-terminal occludin and actin filaments. Phosphorylation of myosin light chain by myosin light chain kinase (MLCK) will cause contraction of the perijunctional actomyosin ring and opening of the tight junction channels.

**Figure 5:**
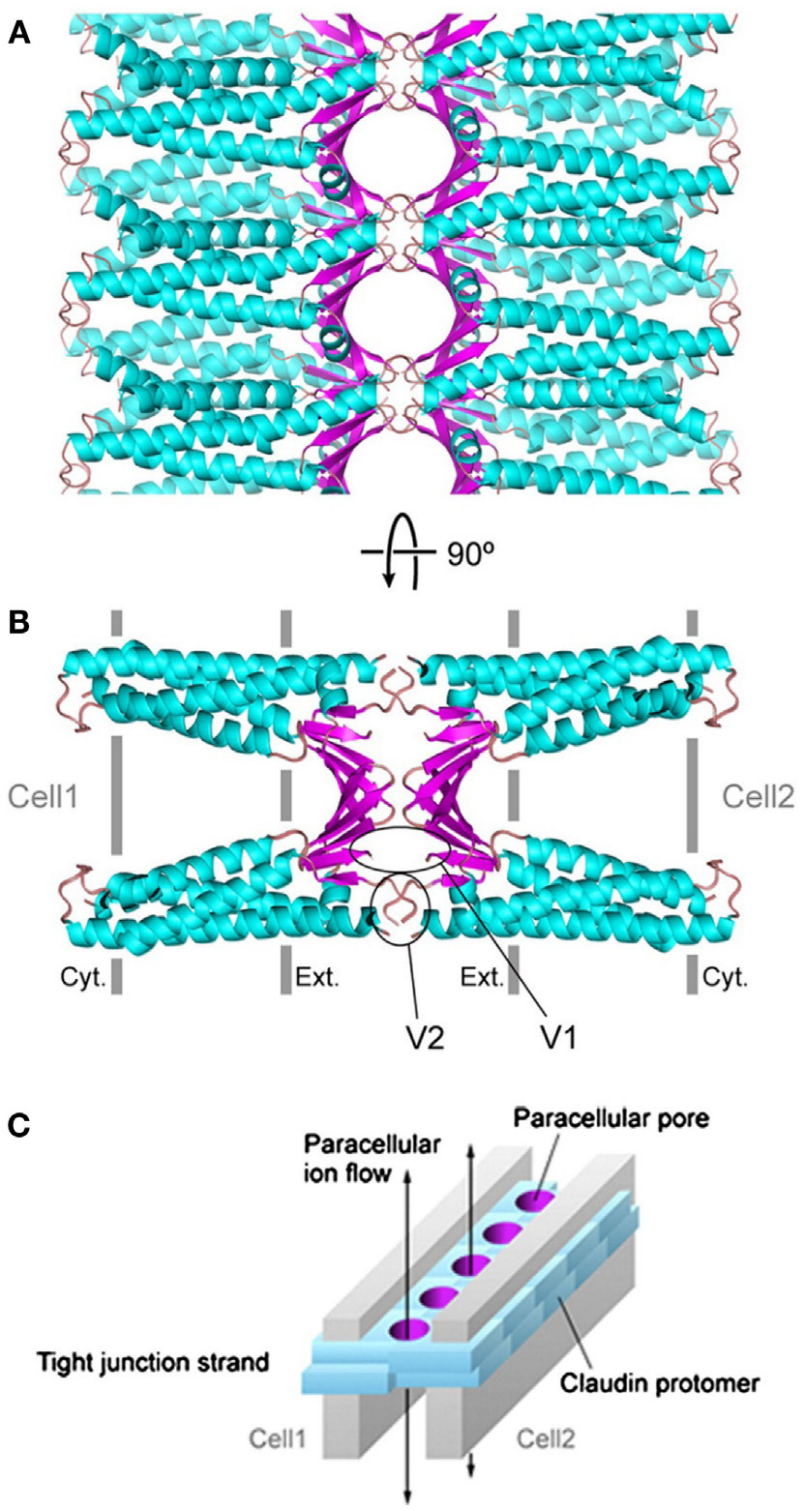
**A model of paracellular claudin-based tight junction channels**. Putative β-barrel-like channels are formed by the extracellular domains (in magenta) of double-layered claudins, whose transmembrane helices are depicted in cyan. The tight junction complex is shown in parallel **(A)** and perpendicular **(B)** views, respectively, from the apical surface of the cells. **(C)** A schematic drawing of claudin-based tight junction channels. The paracellular pores are colored in magenta and double-layered claudins are represented by cyan blocks. Two gray plates indicate two neighboring cell membranes, and the arrows indicate the directions of ions passing through the tight junction channels. The graphs are adopted from the open-access reference (81).

Claudins, occludin, and tricellulin are all membrane proteins with four transmembrane domains, one intracellular and two extracellular loops, and two cytoplasmic tails, whereas the JAM proteins are single-pass transmembrane proteins consisting of two extracellular immunoglobulin (Ig)-like domains, a single transmembrane domain, and a short intercellular C-terminal tail (72) (Figure 4). While the N-terminal tail is generally short, the longer C-terminal tail of claudins consists of a post-synaptic density 95, disk-large, and zonula occludens (PDZ)-binding motif that interacts with the first PDZ domain of ZO-1 (73). The crystal structure of claudins as exemplified by mouse claudin-15 indicates that the four transmembrane segments form a tight four-helix bundle with parts of the two extracellular loops forming a “palm-shaped” structure (80). A model for the architecture of claudin-formed TJ strands in the membrane has been proposed based on the results of crosslinking experiments and electron microscopy (81). In this model, claudins show an antiparallel, double-layer arrangement (Figures 5A,B). The association of claudin double layers in neighboring lateral membranes results in the formation of multiple extracellular β-barrel-like pores parallel with the membrane plane to allow the passage of ions through the paracellular space (Figure 5C).

As for occludin, its two extracellular loops mediate homophilic interactions and permeability to macromolecules. The occludin–ELL domain in the C-terminal tail of occludin is responsible for interacting with the GUK domain of ZO proteins, while the N-terminal tail lacks a defined function (75) (Figure 4). In comparison with claudins and occludin, tricellulin consists of two long tails, with the C-terminal tail containing an occludin–ELL domain that is likely to interact with the GUK domain of ZO proteins (82). JAM proteins also contain a cytoplasmic tail with a PDZ-binding motif that interacts with the third PDZ domain of ZO-1 (83) (Figure 4). The extracellular Ig-like domains of JAM proteins are responsible for homophilic and heterophilic interactions.

The cytoplasmic ZO proteins contain three PDZ domains, a Src homology-3 (SH3) domain, and a GUK domain (78). As stated above, ZO-1 directly interacts with claudins, ZO-2, and JAM-1 through the first, second, and third PDZ domain, respectively. The GUK domain of ZO proteins is known to associate with occludin, while the C-terminal actin-binding region is responsible for bridging with actin, raising the possibility of forming a large protein complex through simultaneous interactions of many TJ proteins with ZO-1 (78). Cingulin is another intracellular plaque protein that is predicted to form a homodimer with globular head and tail at both ends connected by a coiled-coil “rod” domain in the central region. The head of cingulin is known to bind to ZO proteins and the coiled-coil domain interacts with myosin (79). The interactions between the TJ protein complex and cytoskeleton are critical in maintaining and regulating the TJ structure and function, as the mucosal permeability is regulated heavily by the phosphorylation status of myosin light chain, which can be modified by the kinases such as myosin light chain kinase (MLCK) (72, 73) (Figure 4).

### Tight Junctions’ Function in Selective Permeability

Tight junctions are distributed at the apical surface of epithelial and endothelial cells throughout the body in vertebrate animals, including the skin, GI, respiratory, and urogenital tracts as well as the blood vessels (72). TJs are the major determinant of mucosal barrier permeability. Ions, water, and macromolecules pass TJs through either of the two major types of pores. The non-restrictive pathway, also known as the “leak” pathway, allows the transport of macromolecules through large pores with no charge selectivity, while the restrictive or “pore” pathway is only permeable to small ions through pores of ~4 Å in radius with charge selectivity (1, 3, 4). Claudins are mainly responsible for the “pore” pathway, and the two extracellular loops work as an “electrostatic selective filter” to select the size and charge for small pores (Figure 5). The charge selectivity of individual claudins is determined by the net charge of the amino acid residues in the first extracellular loop. For example, claudin-1 is selective for anions, while claudin-2 prefers cations. In the intestinal tract, claudin-1, -3, -4, -5, -8, -9, -11, and -14 decrease paracellular permeability and are regarded as barrier-forming claudins, while claudin-2, -7, -12, and -15 are to increase permeability and generally referred as pore-forming claudins (72, 73).

The non-restrictive “leak” pathway is primarily dependent on occludin as evidenced by an increase in paracellular flux of macromolecules without a noticeable effect on transepithelial electrical resistance (TEER) both *in vitro* and *in vivo* after occludin knock-down (84). JAM proteins also enhance the TJ function by decreasing permeability and facilitating the assembly of occludin to the TJ complex (83). In addition, JAMs can regulate the paracellular barrier for the transmigration of leukocytes from the blood vessel to inflamed sites in response to inflammation (76). It is noteworthy that paracellular water permeability is mainly dictated by claudin-2.

### Modulation of Paracellular Permeability

A number of agents such as cytokines, growth factors, pathogens, probiotics, nutrients, and phytochemicals have been found to impact TJ permeability and mucosal barrier functions through transcriptional regulation and posttranslational modification of TJ proteins (72, 73). Increased expressions of barrier-forming claudins, occludin, and JAM proteins are commonly associated with reduced paracellular permeability and improved barrier function, whereas an elevation in the expression of pore-forming claudins often leads to barrier dysfunction. For example, transforming growth factor (TGF)-β enhances barrier integrity of intestinal epithelial cells by augmenting claudin-1 and -4 expression, while IL-1β, IL-6, and TNF-α increase intestinal cell permeability by increasing claudin-2 expression and/or reducing occludin and ZO-1 expression (72).

Tight junction barrier integrity is also affected by posttranslational modifications of transmembrane and cytoplasmic proteins and associated regulatory proteins. Phosphorylation, glycosylation, and/or ubiquitination of the TJ proteins have a profound impact on barrier permeability. For example, ­claudin-1 is phosphorylated by atypical protein kinase C (aPKC), protein kinase A (PKA), and mitogen-activated protein kinases (MAPK) and dephosphorylated by protein phosphatase 2A (72, 73). Phosphorylation of claudins generally promotes their assembly into the TJ, whereas dephosphorylation often results in the dissociation of claudins from the TJ (72, 73). Similarly, phosphorylation of occludin enhances the barrier function, while dephosphorylation delays the TJ assembly resulting in barrier dysfunction (72, 75).

Besides those proteins involved directly in the TJ assembly, paracellular permeability is also heavily influenced by actin–­myosin filaments that are linked to the TJ proteins. It is well known that up-regulation of MLCK is linked to an increase in the TJ permeability by catalyzing the phosphorylation of myosin light chain, which in turn induces the contraction of actin–myosin filaments and opening of the TJ barrier (85). Both IL-1β and TNF-α are strong inducers of the MLCK gene transcription and activation, resulting increased myosin light chain phosphorylation and TJ barrier permeability (86, 87).

The expression and posttranslational modifications of TJ proteins are influenced by a complicated network of signaling pathways that intertwine with each other. Activation of nuclear factor (NF)-κB signaling by pathogens, pathogen-associated molecular patterns (PAMPs), and proinflammatory cytokines often causes an increase in intestinal epithelial permeability through induction of pore-forming claudin-2 and suppression of barrier-forming claudins such as claudin-1, -3, -4, -5, -7, and -8. On the other hand, activation of TGF-β/SMAD and PPAR-α/γ signaling are generally barrier protective by enhancing claudin-1 and -4 expressions while downregulating claudin-2 (73).

### Implication of Tight Junction Dysfunction in Disease Pathogenesis

The intestinal barrier helps to maintain homeostasis between gut microbiota and the immune system. TJ dysfunction is associated with many enteric disorders such as CD, UC, and celiac disease (3, 72). In CD patients, the expressions of barrier-forming claudin-3, -5, -8, occludin, and JAM-1 are decreased, while pore-forming claudin-2 is significantly increased; and in UC patients, a down-regulation of claudin-1, -4, and JAM-1, and up-regulation of claudin-2, is observed (3, 72). Moreover, increased MLCK expression and activity are evident in both CD and UC patients (85). These factors collectively exacerbate the intestinal paracellular permeability leading to a “leaky gut syndrome.” However, many of these clinical conditions are also accompanied with increase synthesis of proinflammatory cytokines, which are known to cause barrier dysfunction. Thus, it is difficult to determine whether barrier dysfunction is a cause or effect of many of these diseases.

Several enteric pathogens such as *Vibrio cholera*, enteropathic *Escherichia coli*, *Clostridium perfringens* are known to cause diarrhea mainly through disruption of the intestinal barrier function by secretion of exotoxins (72). For example, claudin-3 and -4 are receptors for *C. perfringens* enterotoxin, and the binding of enterotoxin to the extracellular loops of claudins causes internalization of claudins and disintegration of the TJ assembly (88). Early weaning (<3 weeks of age) is known to impair the development of intestinal barrier functions of pigs leading to more pronounced diarrhea (89), and was recently found to lead to reduced expressions of occludin, claudin-1, and ZO-1 in the jejunum (90).

## Regulation of Tight Junction and Mucin Production by Host Defense Peptides

### Association of HDP Expression with Barrier Dysfunction

Along with decreased TJ protein expression, aberrant HDP expression is common in CD and UC patients (91). The expression of Paneth cell α-defensins (HD5 and HD6) is significantly reduced in ileal CD patients, but unaffected in colonic CD patients Z (92). Instead, a reduced expression of HBD-1 and HBD-2 is observed in colonic CD (93). Moreover, induction of cathelicidin LL-37 and HBD-2, -3, and -4 is also reduced in colonic CD relative to healthy subjects (94–97). This lack of HDP induction in CD patients is thought to play a key role in CD pathogenesis as it indicates a lack of intestinal immune response. A deficiency in HD5 and HD6 synthesis is even more pronounced in patients carrying a mutation in the intracellular NOD2 receptor (98), which is expressed by Paneth cells. Consistently, NOD2-knockout mice show a diminished expression of Paneth cell α-defensins known as cryptdins in mice (99). In contrast to CD patients, UC patients display unchanged HD5 and HD6 expressions (92), while LL-37, HBD-2, -3, and -4 are upregulated (94–97). A thin or even absent mucus layer is evident in UC intestinal segments, which causes intestinal inflammation due to direct adhesion and invasion of bacteria to mucosal epithelial cells. Although UC patients produce HDPs, these peptides are not retained in the intestinal tract. Perhaps the most convincing evidence linking the positive role of HDPs in barrier function comes from the studies with cathelicidin (CRAMP)-deficient mice. These mice show delayed recovery of barrier permeability in response to acute disruption of epidermal barrier, albeit with subtle barrier abnormalities in the epidermis (100). Collectively, these lines of evidence suggest a direct impact of HDPs on intestinal barrier function and homeostasis.

### Transcriptional Regulation of Mucins and TJ Proteins by HDPs

Accumulating pieces of evidence suggest a direct involvement of HDPs in regulating the synthesis of mucins and TJ proteins in the intestinal tract. HBD-2 upregulates MUC2, MUC3, but not MUC1 or MUC5AC in human HT-29 colonic epithelial cells (101). MUC2 expression is also enhanced in human Caco-2 colonic epithelial cells in response to HBD-2 (101), and upregulated MUC2 in turn promotes HBD-2 expression (102), suggestive of a positive feedback mechanism between MUC2 and HBD-2. LL-37 also enhances MUC1, MUC2, and MUC3 expressions in HT-29 cells (103, 104) and MUC3 expression only in Caco-2 cells (104). Buforin II, a 21-amino acid HDP isolated from the stomach of an Asian toad (*Bufo bufo garagriozans*), improves intestinal barrier function in weaned piglets challenged with three enterotoxigenic *E. coli* (ETEC) strains (105). Oral administration (twice daily) of buforin II leads to an increase in claudin-1, occludin, and ZO-1 expression in the jejunal segments of *E. coli*-challenged piglets (105). Importantly, buforin II also improves intestinal morphology and growth performance and reduced bacterial shedding in fecal swabs (105). Additionally, administration of a banded krait HDP known as cathelicidin-BF induces ZO-1 expression in the jejunum of healthy mice and also restores LPS-mediated impairment of ZO-1 and intestinal barrier function (106). Furthermore, porcine β-defensin-2 (PBD-2) is capable of restoring the expression of MUC1, MUC2, claudin-1, ZO-1, and ZO-2 as well as the barrier integrity of the colon of DSS-treated mice (107).

Besides direct regulation of the intestinal paracellular permeability, several HDPs also positively influence the barrier effect of the respiratory tract and the skin. LL-37 induces MUC5AC in human NCI-H292 airway epithelial cells (108). LL-37, HBD-3, and S100A7/psoriasin are all able to augment the expression of TJ proteins in human skin keratinocytes (109–111). HBD-3 induces the expressions of all 14 claudins examined, but not occludin or ZO 1–3 in human keratinocytes (110). Similarly, LL-37 dose-dependently enhances the expressions of 11 claudins and occludin in skin keratinocytes, but not JAM 1–3 or ZO 1–3 (109). S100A7 also promotes the expressions of multiple claudins and occludin, but not JAM 1–3 or ZO 1–3 in human keratinocytes (111). Multiple signaling pathways are involved in HDP-induced barrier protein synthesis as detailed below.

## Molecular Mechanisms of HDP Regulation of Barrier Functions

A number of extracellular and intracellular receptors have been reported to be responsible for a range of physiological functions of cathelicidins and defensins in humans and mice. LL-37 and the mouse ortholog (CRAMP) are ligands for P2X purinergic receptor 7 (P2X7), formyl peptide receptor-like (FPRL) 1/2, glyceraldehyde 3-phosphate dehydrogenase, and sequestosome-1/p62, whereas several human and mouse β-defensins bind to CC chemokine receptor 2 (CCR2), CCR6, CXC chemokine receptor 2 (CXCR2), and toll-like receptor 1/2/4 (112, 113). The receptors and signaling pathways by which HDPs induce the expression of mucins and TJ proteins have been studied, but remain elusive in most cases. It is worth noting that, although most published mechanistic studies were based on skin keratinocytes, the following overall conclusions are believed to be applicable to intestinal epithelia as well: (1) HDPs vary greatly in their ability to modulate barrier function, albeit with structural similarities, (2) mucins and TJ proteins are differentially regulated by HDPs, (3) multiple signaling pathways are employed by the same HDP, and (4) receptors appear to be differentially engaged in mediating the induction of mucins and TJ proteins by different HDPs. The current findings are summarized below.

### Signaling Mechanisms of HDP-Mediated Mucin Induction

**Figure 6:**
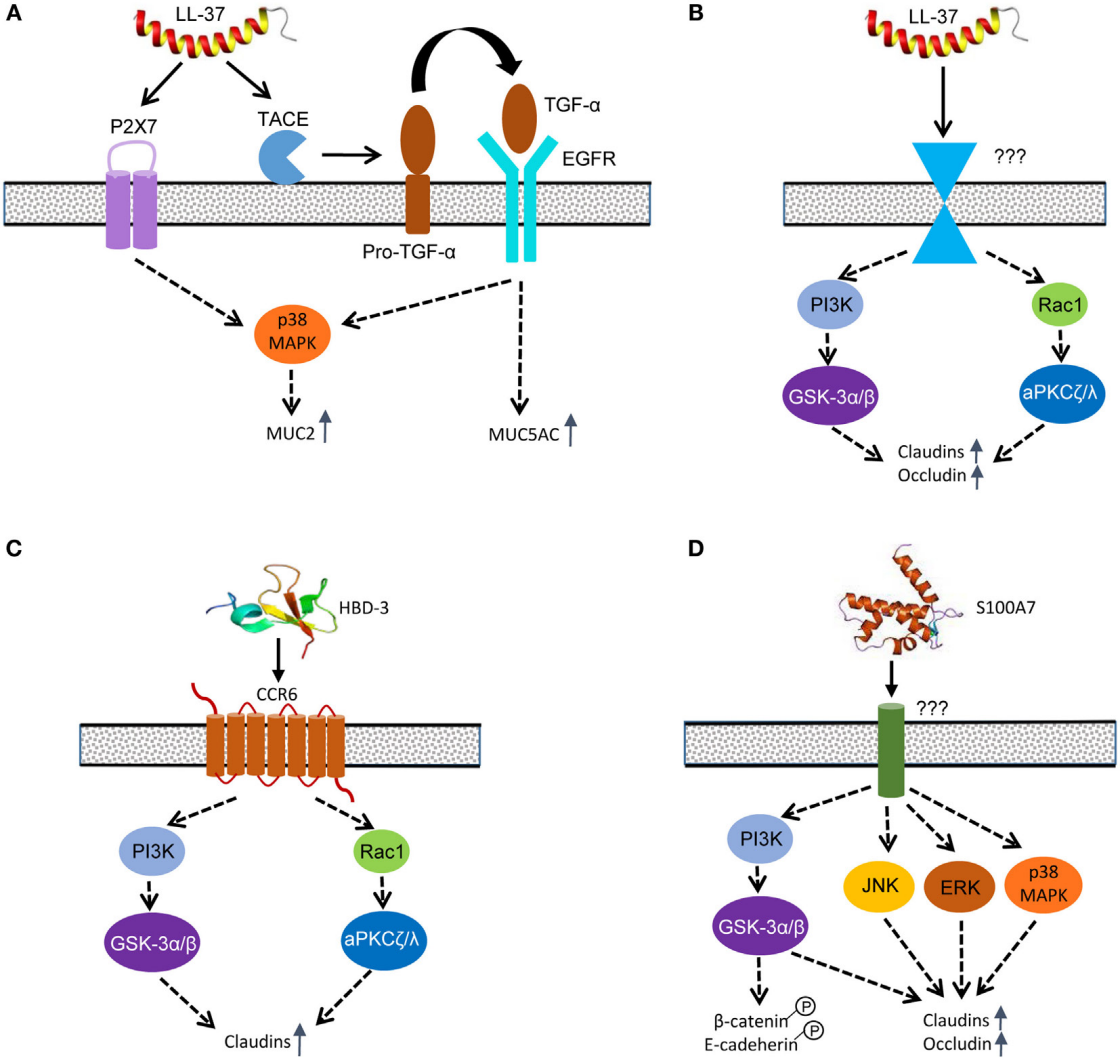
**Host defense peptide-mediated signaling pathways to induce mucins and tight junction proteins in epidermal and intestinal epithelial cells**. **(A)** LL-37 primarily utilizes purinergic receptor P2X7 and transactivates EGFR to mediate MUC2 and MUC5AC induction. **(B)** LL-37 induces the synthesis of multiple claudins and occludin in skin keratinocytes mainly through PI3K-GSK-3α/β and Rac1-aPKCζ/λ pathways; however, the receptor that mediates the effect is currently unknown. **(C)** HBD-3 mainly engages CCR6 to induce claudin synthesis in skin keratinocytes through the PI3K-GSK-3α/β and Rac1-aPKCζ/λ pathways. **(D)** S100A7 enhances the synthesis of claudins and occludin through PI3K-GSK-3α/β and three canonical MAPK pathways. S100A7 also triggers phosphorylation of β-catenin and E-cadherin to enhance the adherens junction. A solid arrow indicates a direct effect, whereas a dashed arrow refers to an indirect action. It is noted that cross-talks among different signaling pathways likely exist, but they have not been reported.

LL-37-induced MUC5AC expression in lung epithelial cells appears to be mediated mainly through transactivation of the EGF receptor (EGFR) (108), although EGFR is not a direct receptor for LL-37 (Figure 6A). Initially, LL-37 triggers the activation of TNF-α-converting enzyme, which in turn cleaves the membrane-bound form of TGF-α, but not heparin binding-EGF. Released TGF-α subsequently interacts with and phosphorylates its receptor, EGFR, which induces MUC5AC gene expression through activation of multiple signaling pathways (108). For LL-37 to induce MUC2 and MUC3 expression in human intestinal epithelial cells, both EGFR and P2X7, but not G-protein-coupled receptors, are involved (104). HBD-2-induced mucin expression in human intestinal epithelial cells is shown to be partially mediated through CCR6 (101). The p38 MAPK, but not extracellular signal-regulated kinase (ERK) or PI3K, is involved in mediating P2X7- and EGFR-activation of MUC2 production in human Caco-2 cells (104).

### LL-37-Mediated TJ Protein Induction

Rac1, aPKCζ/λ, glycogen synthase kinase (GSK)-3α/β, and PI3K are all phosphorylated and activated in human skin keratinocytes in response to LL-37, and blockage of any enzyme with a specific inhibitor results in a substantial reduction in the TEER and a significant increase in the permeability to FITC-dextran (109) (Figure 6B). Rac1 is a small GTPase that functions upstream of aPKCζ/λ as part of the Par3/Par6/aPKCζ/λ polarity complex, which in turn phosphorylates the C-terminal domain of occludin (114) or JAM-1 (115), promoting its assembly into the TJ complex and enhancing the barrier function (116). GSK-3α/β is involved in a number of signaling pathways (117) and is required for induction of occludin and claudin-1 in intestinal and kidney epithelial cells (118). Consistently, LL-37 triggers phosphorylation and activation of GSK-3α/β at Tyr 216 and Tyr 279 in human keratinocytes at 1–2 h after exposure, leading to the improvement of epidermal barrier function (109).

PI3K functions upstream of GSK-3, and the PI3K signaling cascade has been implicated in both the degradation and stimulation of TJ barrier function depending on the stimulating agent (119). PI3K is quickly phosphorylated within 30 min in human keratinocytes upon stimulation with LL-37 (109). In intestinal cells, PI3K plays a key role in directing proper occludin localization and subsequent tightening of epithelial barrier function in response to prostaglandins (120). Inhibition of PI3K in porcine ischemia-injured ileal mucosa attenuates the ability of prostaglandin to recover proper barrier function. In rat Con8 mammary epithelial cells, glucocorticoid recruits Ras and the p85 subunit of PI3K to the TJ complex and increases barrier function (121). However, the specific cellular receptor(s) mediating LL-37-induced TJ protein expression remain unknown and warrant further investigation.

### HBD-3-Mediated TJ Protein Induction

Although HBD-2 is capable of inducing mucin expression (101), only HBD-3 triggers the synthesis of multiple TJ proteins (110). CCR6 has been shown to be primarily responsible for HBD-3-induced enhancement of barrier integrity in epidermis (110). Similar to LL-37, HBD-3 is also capable of phosphorylating and activating Rac1, aPKCζ/λ, PI3K, and GSK-3α/β in similar kinetics in human skin keratinocytes (110) (Figure 6C). Of note, toll-like receptors, PKA, and MAPK pathways are not involved in mediating HBD-3-induced barrier function improvement (110). Although HBD-1, HBD-2, and HBD-4 fail to alter the epidermal permeability, they also have weak activities in activating Rac1, aPKC, GSK-3, and PI3K (110), suggesting those pathways may not be solely devoted to the TJ functions. It is important to note that, although similar in the tertiary structure, only HBD-3, but not HBD-1, -2 or -4, has the ability to trigger the induction of TJ proteins (115).

### S100A7-Mediated TJ Protein Induction

The role of GSK-3α/β and MAPK in human epidermal barrier function mediated by S100A7 has been studied (111) (Figure 6D). GSK-3α/β is phosphorylated and activated at Tyr 216 and Tyr 279 within 30 min following exposure of human keratinocytes to S100A7/psoriasin. Specific inhibition of GSK-3 activation abolishes induction of claudins and epidermal TEER by S100A7 (111). Because β-catenin is regulated directly by GSK-3 (117), S100A7 is revealed to phosphorylate and activate β-catenin, which is vital to the assembly of adherens junctions. E-cadherin, another essential component of the adherens junction complex, is also phosphorylated by S100A7 (111), suggesting that, besides the TJs, S100A7 also improves the assembly of adherens junctions.

The MAPK pathway includes three canonical signaling cascades that consist of ERK, c-Jun N-terminal kinase (JNK), and p38 (122). Collectively, they are critical to many important physiological processes ranging from cell division and differentiation to stress and immune responses. Unlike HBD-3, S100A7 is capable of activating all three canonical MAPK cascades (111). ERK is quickly phosphorylated in 2 min in human skin keratinocytes following exposure to S100A7, while JNK and p38 MAPK are also phosphorylated in 30 min. Inhibition of individual MAPK signaling cascades leads to a substantial reduction in claudin induction and epidermal TEER (111), implying that all three major MAPK pathways are required. However, the involvement of any specific receptors or other signaling pathways remains to be studied.

## Role of HDPs in Intestinal Mucosal Homeostasis, Immune Defense, and Growth Performance

One of the major functions of the intestinal epithelium is to act as a barrier against the invasion of microorganisms. This task is especially difficult considering that the intestinal mucosa is colonized by over 1013 microorganisms (123), with the majority being commensal bacteria that are beneficial to the host through their ability to improve digestion, absorption, and vitamin synthesis while also limiting pathogen growth (124). The two most dominant bacterial phyla present in the intestinal tract of humans and mice are Gram-negative Bacteriodetes and Gram-positive Firmicutes, which together comprise about 70–80% of the total bacteria present (125). Commensal bacteria are vital to the development of normal intestinal morphology and immune system (126, 127). While commensal bacteria are beneficial to the host under homeostatic conditions, a state of dysbiosis, or imbalance of the microbial community, leads to inflammation and disturbed epithelial homeostasis. This is particularly seen in the CD patients in which the host immune system displays increased activation against commensal microbiota.

The intestinal epithelium continuously monitors resident microbes through interactions between pattern recognition receptors (PRRs) and microbe-associated molecular patterns (MAMPs). Activation of PRRs stimulates the synthesis and release of HDPs and mucins from intestinal cells (126, 127). A large amount of HDPs secreted from Paneth cells and enterocytes are retained in the mucus layer to create a strong barrier against bacterial invasion (128). Studies with HDP-knockout and -transgenic mice have illuminated the role of HDPs in intestinal homeostasis and immune defense. Knockout of the mouse cathelicidin CRAMP gene causes exaggerated colitis in the colon of mice, and the disease symptoms are further exacerbated following DSS treatment (129). Adoptive transfer of bone marrow cells from the wild-type mice to CRAMP-knockout mice alleviates DSS-induced colitis (129). Mice carrying the transgene for HD5 show an augmented ability to fight off orally challenged *S. enterica* serovar Typhimurium (130). Conversely, matrix metalloproteinase 7 (MMP7)-knockout mice with a deficiency in producing biologically active enteric defensins display reduced capacity to clear enteric pathogens (131). Furthermore, a comparison between those two complementary mouse models has revealed dramatic defensin-dependent reciprocal shifts in the intestinal bacterial composition. In comparison with wild-type mice, Firmicutes are reduced and Bacteriodes are increased in small intestine of HD5-transgenic mice, while the opposite is true with MMP7-deficient mice (125, 132). Moreover, overexpression of HD5 in mice causes a significant loss of segmented filamentous bacteria in the distal small intestine and a reduced presence of Th17 cells in the lamina propria (132), suggesting clearly that enteric HDPs represent a critical factor in shaping the microbiota composition and inflammatory status of the GI tract.

Multiple studies have highlighted the beneficial effects of direct feeding of HDPs on growth, intestinal morphology, and immune status in pigs (133, 134). Dietary supplementation of an *E. coli*-producing bacteriocin, colicin E1, significantly improved weight gain and feed efficiency of ETEC-challenged weanling pigs in a 4-day trial, relative to the control pigs (135). Colicin E1 inclusion also reduced the *E. coli* titers in both the fecal and ileal samples as well as the incidence and severity of diarrhea (135). Moreover, the expression levels of proinflammatory cytokines (IL-1β and TNF-α) were reduced in the ileum of pigs in response to colicin E1 feeding (135). Similarly, feeding a recombinant silkworm HDP, cecropin A/D, improved growth and feed efficiency and reduced diarrhea incidence in ETEC-challenged weanling pigs, without an obvious impact on intestinal morphology or nitrogen/energy utilization over a period of 6 days (136). Dietary inclusion of a recombinant fusion HDP derived from bovine lactoferrin also enhanced growth performance and decreased the incidence of diarrhea in ETEC-challenged piglets over a 21-day period (137). Across five different commercial farms, feeding a mixture of four recombinant HDPs, including lactoferrin, cecropin, defensin, and plectasin resulted in an enhancement of growth and feed efficiency and a reduction in diarrhea incidence in normally reared weanling pigs (138). Parallel to the studies above, supplementation of a synthetic HDP (AMP-A3 or P5) improved nutrient digestibility, intestinal morphology, and growth performance of normally reared weanling pigs in a 4-week trial, without affecting serum concentrations of IgA, IgG, or IgM (139, 140). Additionally, AMP-A3 and P5 appeared to reduce the titers of potentially harmful *Clostridium* spp. and coliforms in the ileum, cecum, and feces (140). Feeding a combination of two HDPs and a probiotic yeast led to an improvement in intestinal morphology and feed efficiency of piglets challenged with deoxynivalenol, a mycotoxin commonly found in grains (141, 142). In most trials above, HDPs performed indistinguishably with in-feed antibiotics in promoting growth, feed efficiency, and intestinal morphology (133).

The beneficial effects of direct feeding of HDPs are not limited to pigs. Supplementation of AMP-A3 to broiler chickens resulted in an increase in weight gain and feed efficiency over control birds, which was comparable to the birds fed avilamycin, an in-feed antibiotic (143). Intestinal morphology was also improved in broilers as measured by increased villus heights and villus height:crypt depth ratios in the small intestine (143). Similar to the results in pigs, broilers also displayed an improvement in nutrient utilization and a reduction in *Clostridium* spp. and coliforms in the intestinal tract (143). Supplementation of a yeast broth containing recombinant cecropin A/D improved intestinal morphology and nutrient utilization, with a tendency to enhance growth performance of broiler chickens in a 4-week trial (144). Cecropin A/D inclusion also reduced the total aerobic bacterial counts in both the jejunal and cecal contents of 42-day-old chickens (144). Collectively, these animal results have suggested the beneficial effects of HDP feeding, justifying dietary supplementation of HDPs as an antibiotic-alternative strategy in growth promotion and disease control.

However, because of HDP’s proneness to enzymatic degradation and high production costs with either the synthetic or recombinant form, it may not be biologically efficient and economically effective for direct supplementation of HDPs in animal diets. Recently, several classes of small-molecule compounds, such as butyrate, have been found to induce HDP synthesis and enhance bacterial clearance in humans, chickens, pigs, and cattle without triggering inflammatory response (145–151). Dietary supplementation of these simple HDP-inducing compounds or their combinations may prove to be an alternative, cost-effective approach to antibiotics for livestock applications (152). However, the efficacy of these HDP-inducing compounds in promoting growth, intestinal health, and microbiota balance is yet to be demonstrated in animal trials.

## Concluding Remarks

A comprehensive understanding of intestinal barrier function and its regulation is paramount to ensuring the sustainability of the food animal industry because disruption of the barrier results in disease states and decreased production efficiency (6). With potent antimicrobial and immunomodulatory properties, HDPs are further revealed to hold a new capacity to directly regulate barrier function. Aberrant synthesis of epithelial HDPs often leads to barrier dysfunction, and the diseases with impaired barrier integrity are commonly associated with reduced HDP synthesis, raising the possibility of treating barrier dysfunction with HDPs. However, a number of questions remain before HDP-based therapies can be devised for augmentation of intestinal barrier function, animal health, and production efficiency.

On the one hand, several structurally diverse HDPs (e.g., cathelicidins, defensins, and S100A7) have a similar ability to induce the expression of mucins and TJ proteins in both epidermal and intestinal epithelial cells. On the other hand, certain structurally similar HDPs (e.g., HBD-3 vs. HBD-1, -2, and -4) behave quite differently in their capacity to induce TJ proteins. Only HBD-3, but not other HBDs, has the capacity to enhance the barrier effect (110). Structure–activity relationship studies of HDPs may reveal whether there is an optimal physicochemical or structural feature for maximal induction of mucins and TJ proteins.

A number of different extracellular and intracellular receptors have been identified for human HDPs to mediate different physiological functions. However, the identities of the receptors utilized by different classes of HDPs to regulate paracellular permeability remain largely unclear in most cases. There are a number of questions on the signaling mechanisms of HDP-mediated barrier function that need to be answered. For example, what are the major receptors involved in HDP-induced synthesis of mucins and TJ proteins in humans? Do the same set of receptors that are utilized by human HDPs work similarly in the livestock species? Are there any new, unidentified receptors specific for regulation of barrier function? Besides the Rac1-aPKC, PI3K-GSK-3, and MAPK pathways, what are other major signaling pathways that mediate the barrier effect? How do these pathways cross-talk with each other? What are those major transcription factors that are required for induction of different mucin and TJ proteins? How and whether are mucins and TJ proteins differentially regulated? Do epithelial cells on epidermis, GI, respiratory, and genitourinary tracts engage in different receptors and signaling pathways?

Besides the abundance of TJ proteins, both posttranslational modifications of TJ proteins and the status of associated actomyosin ring have a strong impact on barrier permeability. Many agents are shown to alter the barrier function through phosphorylation of certain TJ proteins or through activation of MLCK, which in turn phosphorylates myosin light chain and causes contraction of the perijunctional actomyosin ring and opening of the paracellular pores. It will be important to examine whether and how HDPs influence posttranslational modifications of TJ proteins as well as the transcription and activity of MLCK.

Nevertheless, it is exciting to reveal a direct involvement of HDPs in barrier function and the potential of HDPs in enhancing gut health and animal performance. Additional studies along this line may someday turn the HDP-based therapies into reality. Although administration of synthetic peptides may be feasible in human medicine, it is cost-prohibitive in the livestock industry. Supplementation of exogenous recombinant HDPs or dietary compounds with the capacity to induce the synthesis of HDPs in the GI tract has emerged as a cost-effective strategy in antimicrobial therapy and may have potential to replace antibiotics in food animal production.

## Conflict of Interest Statement

The authors declare that the research was conducted in the absence of any commercial or financial relationships that could be construed as a potential conflict of interest.

## Abbreviations

aPKC, atypical protein kinase C; CCR2, CC chemokine receptor 2; CD, Crohn’s disease; DSS, dextran sulfate sodium; EGF, epidermal growth factor; EGFR, EGF receptor; ERK, extracellular signal-regulated kinase; ETEC, enterotoxigenic *E. coli*; GI, gastrointestinal; GSK-3, glycogen synthase kinase 3; GUK, guanylate kinase; HBD, human β-defensin; HDPs, host defense peptides; JAM, junction adhesion molecule; LPS, lipopolysaccharides; MAMPs, microbe-associated molecular patterns; MAPK, mitogen-activated protein kinase; MBD, mouse β-defensin; MLCK, myosin light chain kinase; MUC, mucin; P2X7, P2X purinergic receptor 7; PAMPs, pathogen-associated molecular patterns; PBD, porcine β-defensin; PDZ, post-synaptic density 95, disk-large, and zonula occludens; PI3K, phosphatidylinositol-3-kinase; PKA, protein kinase A; PKC, protein kinase C; PRRs, pattern recognition receptors; SEA, sea urchin sperm protein, enterokinase and agrin; TACE, TNF-α-converting enzyme; TEER, transepithelial electrical resistance; TGF, transforming growth factor; TJ, tight junction; TR, tandem repeat; UC, ulcerative colitis; VWF, von Willebrand factor domain; ZO, zonula occludens.
